# Top-Down Preparation of Nanoquartz for Toxicological Investigations

**DOI:** 10.3390/ijms232315425

**Published:** 2022-12-06

**Authors:** Chiara Bellomo, Cristina Pavan, Gianluca Fiore, Guillermo Escolano-Casado, Lorenzo Mino, Francesco Turci

**Affiliations:** 1 Department of Chemistry, University of Turin, 10125 Turin, Italy; 2 “G. Scansetti” Interdepartmental Centre for Studies on Asbestos and Other Toxic Particulates, University of Turin, 10125 Turin, Italy; 3 Louvain Centre for Toxicology and Applied Pharmacology, Université catholique de Louvain, 1200 Brussels, Belgium; 4 Nanostructured Interfaces and Surfaces Interdepartmental Centre, University of Turin, 10125 Turin, Italy

**Keywords:** silica, quartz, nanoparticle, silanol, crystallinity, fracturing, milling

## Abstract

Occupational exposure to quartz dust is associated with fatal diseases. Quartz dusts generated by mechanical fracturing are characterized by a broad range of micrometric to nanometric particles. The contribution of this nanometric fraction to the overall toxicity of quartz is still largely unexplored, primarily because of the strong electrostatic adhesion forces that prevent isolation of the nanofraction. Furthermore, fractured silica dust exhibits special surface features, namely nearly free silanols (NFS), which impart a membranolytic activity to quartz. Nanoquartz can be synthetized via bottom-up methods, but the surface chemistry of such crystals strongly differs from that of nanoparticles resulting from fracturing. Here, we report a top-down milling procedure to obtain a nanometric quartz that shares the key surface properties relevant to toxicity with fractured quartz. The ball milling was optimized by coupling the dry and wet milling steps, using water as a dispersing agent, and varying the milling times and rotational speeds. Nanoquartz with a strong tendency to form submicrometric agglomerates was obtained. The deagglomeration with surfactants or simulated body fluids was negligible. Partial lattice amorphization and a bimodal crystallite domain size were observed. A moderate membranolytic activity, which correlated with the number of NFS, signaled coherence with the previous toxicological data. A membranolytic nanoquartz for toxicological investigations was obtained.

## 1. Introduction

Respirable crystalline silica (RCS) is the fraction of silica dust with an aerodynamic diameter ≤4 µm. Occupational exposure to RCS is associated to several pathologies, including silicosis, lung cancer, and autoimmune diseases [1,2]. Exposure to RCS dusts is currently the main cause of occupation lung cancer in the world. Due to its ubiquitous nature, silica is extensively used in industrial productions and processes, in both crystalline and amorphous forms [3,4]. RCS is commonly made of quartz particles but more rarely made from cristobalite or a mixture of the two polymorphs. Quartz is classified as carcinogenic to humans (group 1) by the International Agency for Research on Cancer (IARC) [5]. However, different sources of quartz often exhibit variability in their toxic effects [6]. Such variability has been associated with the structural and surface modifications induced by the production and processing of quartz sands [7].

Mechanical processes, including milling, sandblasting, cutting, and polishing, of quartz-containing stones and composites, induce the fracturing and disorganization of the uppermost atomic surface layers of the crystals, which lose the long-range order of the quartz lattice [8]. This forms a new layer, which is subjected to chemical and structural rearranging, in a dynamic process of surface reconstruction that generates silanols (≡Si–OH) upon reaction with molecular water, possibly spaced by siloxane bridges (≡Si–O–Si≡) [9]. It has been shown that fractured quartz has a higher capacity to induce cell membrane damage and initiate lung inflammatory reactions in rodents than non-fractured quartz with unaltered surfaces [10,11]. Upon fracturing, quartz originates a specific population of surface silanols, namely the nearly free silanols (NFS). NFS were identified on fractured and disordered surfaces generated during the milling process, exhibiting a peculiar intersilanol distance (4 to 6 Å) that is higher than that of the strongly interacting silanols (ca. 2.5 Å) on ordered surfaces [11]. Such a distance is not sufficient to describe them as isolated silanols, which are farther from each other by more than 6 Å and clearly distinguishable by the vibrational analysis of the O-H stretching mode. The peculiar nature of this silanol family allows NFS to specifically interact with the phospholipids that make up the cellular membranes [12], and this mechanism proved to cause the activation of the inflammatory response induced by quartz. NFS were observed on both crystalline and amorphous silica and are believed to be related to the acute inflammation and cytotoxic effects that have been observed for quartz [13] and fumed amorphous nanosilica [14,15].

**Figure 1:**
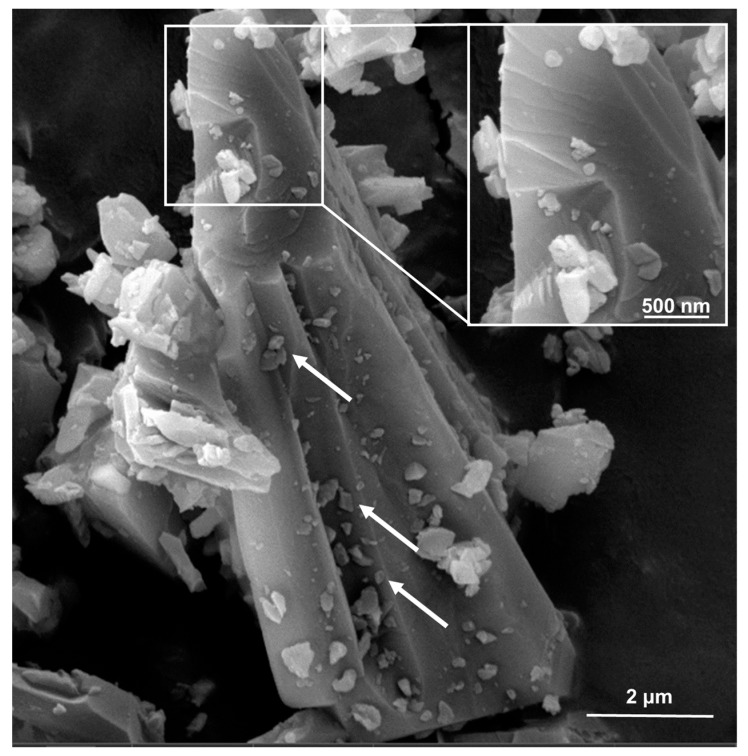
Scanning Electron Microscopy (SEM) micrographs of a respirable crystalline silica dust of industrial origin (cQ-f, in this work). Nanometric particles (highlighted by the white arrows) adhere on the surface of micrometric particles.

In addition to the inherent structural differences that can be observed among silica samples of different origins, different milling conditions may dramatically affect the comminution efficiency and the homogeneity of the particle size. In industrial settings, the limited possibility to control particle size distribution during comminution is even more prominent [16,17,18]. Electron images of milled quartz dusts commonly show the association of micrometric, submicrometric, and nanometric crystals, which firmly adhere to larger particles due to electrostatic interactions (Figure 1).

While the toxicological properties of micrometric quartz dust have been largely investigated in the past several decades [5,19], very little is known about the specific physico-chemical properties of the nanometric fraction and its possible contribution to the overall toxicity of RCS. Even if the relative mass of the nanometric fraction is often negligible with respect to the whole dust, the nanometric particles might outnumber the micrometric particles, de facto generating a nanomaterial, according to the most recent recommendation of the European Commission (EC) on the definition of a nanomaterial [20], i.e., >50% of the particles are <100 nm on a per number basis. It is not possible to exclude that nanoparticles may show a higher chemical and toxicological activity than micrometric particles and bulk material. In fact, the decrease in particle size results in an increase in the specific surface area, curvature, and number of surface active sites [21,22]. These factors may influence the interactions with the external environment, including biomolecules, and an increase in particle surface reactivity may impart higher toxic effects and a different mechanism of action (MoA) to the nanoparticles, with respect to larger particles. The French Agency on Food, Environmental and Occupational Health and Safety (ANSES), recently raised concerns about the possible role of crystalline nanosilica in the toxic activity of fractured quartz dust [23]. As the amount and the crystalline nature of a nanometric fraction might vary, the investigations devoted to address the properties and the toxicity of this fraction were scarce and non-conclusive, mostly due to the difficulty in obtaining a quartz dust of nanometric size. A recent review on the pulmonary toxicity of silica evidenced the general lack of a solid investigation on nanoquartz health effects [24]. Specifically, the authors were able to find only one study on nanoscale and fine-quartz particles in rats [25]. Warheit and coworkers challenged the hypothesis that nanometric particles are always more hazardous than micrometric particles of the same compound, but the study used a synthetic quartz prepared with a bottom-up approach. As this nanoquartz was obtained through hydrothermal crystal growth, its surface properties are likely to differ from the nanometric fraction of a quartz dust generated by fracturing. A different approach was followed by Mischler and coworkers, which used a multi-cyclone sampling array to segregate the finest fraction of a commercial quartz flour. However, their approach relied on the availability of large quantities of quartz dust, which introduced uncertainties for the purity, crystallinity, and occurrence of the accessory mineral phases that are always present in natural samples [26]. A few other studies tried to address the effect of size on the toxicity of crystalline silica [27,28,29], but none of them detailed the production method or the physico-chemical bulk and surface characteristics of the nanosilica. This renders these pioneer works not suitable for drawing conclusions on the mechanics of nanoquartz toxicity.

Among the available approaches, we opted for planetary ball milling. Ball milling energetics and grinding efficiency depend on several factors, including grinding time [30], the frequency of oscillation or rotational speed [31,32], the type of milled material [33], and the size, number, density, and hardness of the milling balls [16,34]. Moreover, different stress forces are imparted by different types of mills. While a vibrational mill reduces the particle size mainly through impact and particle fracturing, a planetary mill operates through friction and particle abrasion [35,36]. Milling forces and effects can be further modulated by adding dispersant agents. When a liquid dispersant, e.g., water or a more volatile solvent, such as isopropanol, is added to the milling mixture (wet milling), a better dispersion of the particles and a more uniform particle size distribution is achieved, in contrast to dry milling. Indeed, the presence of a dispersing agent prevents the caking of the material and limits the formation of the aggregates that may hamper an effective and homogeneous reduction to a uniform size. By lowering the energy transfer from the milling balls to the particles, wet milling also better preserves the crystal structure of the pristine material [37].

To produce a sample that is relevant for the toxicological assessment of crystalline nanosilica, several key parameters have to be considered, including the surface chemistry, the relative amount of the nanometric fraction, the degree of amorphization induced in nanoquartz, and the agglomeration/deagglomeration processes. Specifically, the strong electrostatic interaction that is established between micrometric and nanometric particles renders it virtually impossible to achieve the complete separation and quantification of the two fractions. Furthermore, the comminution procedure induces strain, stress, and amorphization of the quartz particles. The finer particles are, thus, expected to partially lose the long-range order of their crystal structure and exhibit amorphization. Pioneer studies described the first surface atomic layers of ground quartz crystal and showed a partial loss of the long-range order that characterizes well-formed quartz crystals [38]. This surface layer, the so-called Beilby layer [39], is less organized than the underlying crystalline bulk and is rich in NFS, which impart inflammatory properties [11]. Lastly, the tendency toward the agglomeration of these particles can affect the reactivity of surface sites, increase the effective aerodynamic size of agglomerates, and modulate nanoparticle uptake and translocation [40,41].

Although mechanical processing of silica and silica-based materials represents one of the most relevant industrial processes that may accidentally produce silica nanoparticles at the workplace and potentially expose workers to nanomaterials, studies that investigate the chemical characteristics and surface activity of nanoquartz particles generated during milling are not currently available. To fill in this gap, a highly pure synthetic quartz in micrometric size, well-characterized in previous works [10,42], was ball milled in dry and wet conditions, incrementing the milling times and rotational speeds, to obtain a nanometric quartz. Particle and crystallite size distributions, crystalline/amorphous ratios, surface NFS numbers, and membranolytic activities were measured for all the samples and compared with the pristine materials. Moreover, the agglomeration and deagglomeration mechanisms were investigated for the dispersion/separation behavior of quartz nanoparticles.

## 2. Results and Discussion

### 2.1. High-Energy Ball Milling Is an Effective Tool for Top-Down Generation of Nanoquartz

To prepare quartz particles in a nanometric size, a two-step milling approach was adopted.

**Figure 2:**
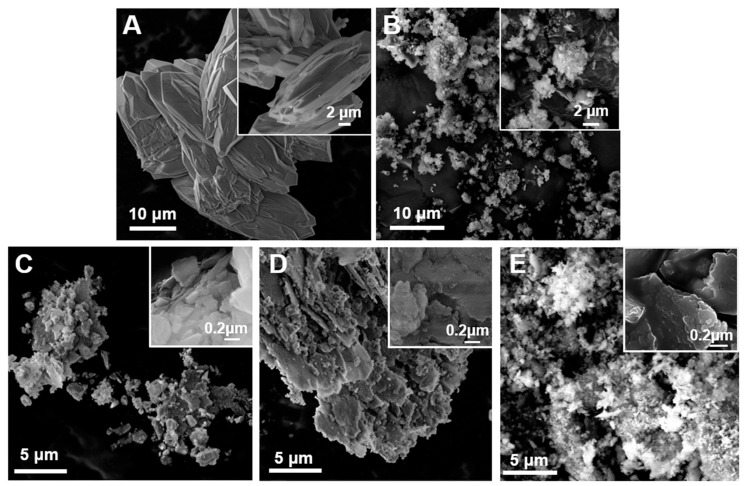
Field emission SEM (FESEM) micrographs at low and high magnification of synthetic as-grown quartz (gQ, (**A**)), fine-fractured quartz (gQ-ff, (**B**)), and nanoquartz samples (gQ-n1, gQ-n2, and gQ-n3, (**C**), (**D**), and (**E**), respectively) obtained by ball milling.

We prepared nanoquartz (gQ-n), starting from a high-pure synthetic as-grown quartz (gQ, 99.9% SiO_2_ from previous characterizations and EDS analysis in Figure S4), which was micrometric in size [10,42]. The as-grown quartz showed regular and smooth surfaces and an average crystal size of 20–30 µm, and no nanometric or submicrometric fraction was observed (SEM micrographs, Figure 2A). Consistent with its micrometric size, a surface area as low as 0.1 m^2^/g was measured by Kr-BET (Table 1). Micrometric gQ crystals were milled in the air for 1 h at 450 rpm rotational speed. A finely fractured quartz dust (gQ-ff), which showed an SSA of 10 m^2^/g and particles with irregular morphology and size, was obtained (Figure 2B) and used as the precursor material for the nanoquartz preparation (see Table 1).

Water was selected as the dispersant, to minimize the alterations of the quartz surface properties and the etherification of the silanols that could occur when organic solvents, e.g., methanol and ethanol, are used [43]. Moreover, the use of a wet step is consistent with the standard procedures used in conventional workplace application (i.e., wet cutting or polishing of quartz containing composites). Thus, the secondary wet-milling steps were designed to induce a four-to-six times increase in the SSA, with respect to gQ-ff (Table 1). By using milling balls with a smaller diameter (2 mm) and by increasing the rotational speed and the milling time up to 550 rpm and 2 h, respectively, the SSAs of the milled quartzes increased by up to 55 m^2^/g in the following order: gQ-ff < gQ-n1 < gQ-n2 < gQ-n3. Despite the loss in energy transfer from ball to sample, the use of a higher number of smaller milling balls generally increases the comminution efficiency, by increasing the number of collisions. Both the milling speed (gQ-n1 vs. gQ-n2) and the milling time (gQ-n2 vs. gQ-n3) strongly affected the efficiency of the comminution. FE-SEM analysis of nanoquartz (Figure 2C–E and Figure S1) showed micrometric lamellar clusters made up of nanometric and submicrometric particles. The number of residual micrometric crystals was negligible. Some isolated submicrometric particles were occasionally detected in gQ-n1 but were no longer visible in gQ-n2 and gQ-n3.

### 2.2. Current Top-Down Approach Mostly Preserves Crystallinity in Nanoquartz

**Figure 3:**
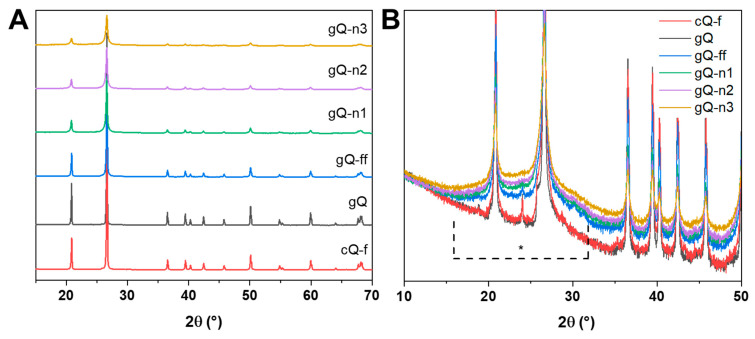
Crystallinity of the nanoquartz samples (gQ-n1, gQ-n2, and gQ-n3) compared with the as-grown micrometric quartz (gQ), the fine-fractured quartz (gQ-ff), and a mined fractured quartz used as reference (cQ-f). Diffractograms obtained by XRPD analysis in the 15–70° region (**A**). Magnification of the diffractograms in the 10–50° region showing the amorphous broad signal (**B**).

High-energy milling may affect material crystallinity. Moreover, micrometric quartz particles, when fractured, show an amorphous surface layer that coexists with the crystalline bulk [38,39]. The crystallinity of gQ-ff and the nanoquartz samples (gQ-n1, gQ-n2, and gQ-n3) was investigated by XRPD and HR-TEM analysis. Rietveld refinement of the diffractograms was carried out to quantify the crystallite size range and amorphous content, with respect to the starting material (gQ). The results of the XRPD analyses are reported in Figure 3. All the nanoquartz samples showed crystallographic patterns in which the position of the diffraction peaks was largely preserved, with regard to pristine quartz (gQ) (Figure 3A). Reductions in the intensity and broadening of the peaks were observed for the gQ-ff and gQ-n samples. A broad signal centered at 25° (2θ range) (Figure 3B) suggests the generation of an amorphous phase for the gQ-ff and gQ-n samples. This is consistent with the partial amorphization of quartz due to ball milling [44].

**Figure 4:**
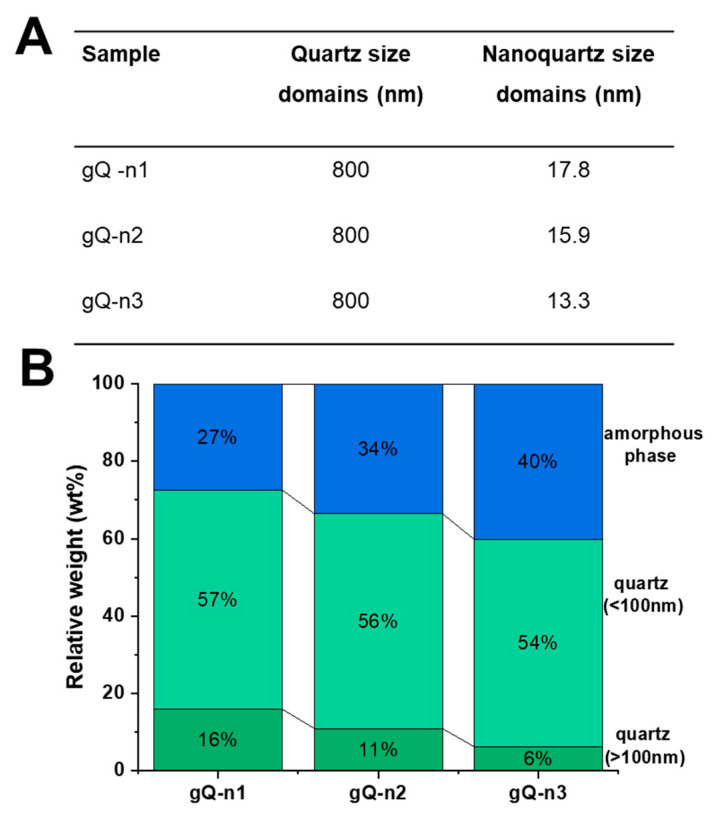
Crystallite size domains (**A**) and relative numbers (wt.%, (**B**)) of submicron (>100 nm), nano (<100 nm), and amorphous phases of nanoquartz samples (gQ-n1, gQ-n2, and gQ-n3), calculated by Rietveld refinement of XRPD diffractograms.

To explore the characteristics of the quartz particles obtained with this mechanism of comminution, Rietveld refinement was performed on the diffractograms of the gQ-n samples. All three nanoquartz particles showed a bimodal size distribution of the crystallite domains, which evidenced the presence of both submicrometric (ca. 800 nm) and nanometric (<100 nm) crystallites (Figure 4A) in variable proportions. As a planetary milling mechanism promotes the formation of smaller particles by inducing the abrasion of the larger crystals, rather than fracturing them by inelastic collision, we speculated that the observed bimodal distribution of crystallite size may be due to this friction-like fracturing mechanism. A planetary ball mill differs from other types of comminution apparatuses, such as a mixer miller, which imparts rapid acceleration and high kinetic energy to the balls and sample particles and promotes fracturing mainly by inelastic collisions [36]. 

**Figure 5:**
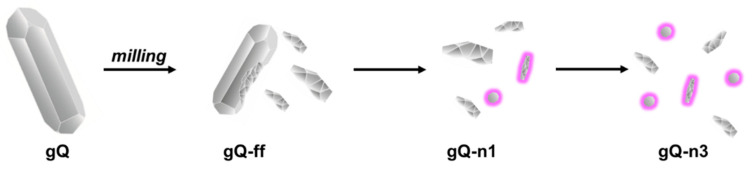
Mechanism of quartz fracturing induced by planetary milling in a wet environment. Prolonged millings cause abrasion of the larger crystals, promoting the formation of nanoparticles, and induce amorphization of the nanoquartz fraction, highlighted with a purple halo. Size of crystallites is not in scale.

The increase in the content of the amorphous phase paralleled the increase in the intensity of the milling energetics (i.e., rotational speed and operational time) (Figure 4B), indicating that mechanical stress induces quartz amorphization, from 27 to 40 wt.% for gQ-n1 and gQn-3, respectively. Along with the increase in the amorphous content, the relative weight of the submicrometric domains also progressively reduced from 16 to 6 wt.%, while the content of the nanometric domains was rather constant, ranging from 57 to 54 wt.% for gQ-n1 and gQn-3, respectively (Figure 4B). This suggests that the ball milling procedure adopted here is effective toward the submicrometric crystallite domains and generates nanometric crystallites. However, the relative content in the nanometric domains appears constant or even slightly reduced by milling, which suggests that the generation of nanometric crystals might be followed by the conversion of these domains into amorphous particles, with a constant net result. This mechanism, which is illustrated in Figure 5, is likely to occur in our system, because nanometric crystallites are more easily subjected to thermal amorphization than larger domains.

**Figure 6:**
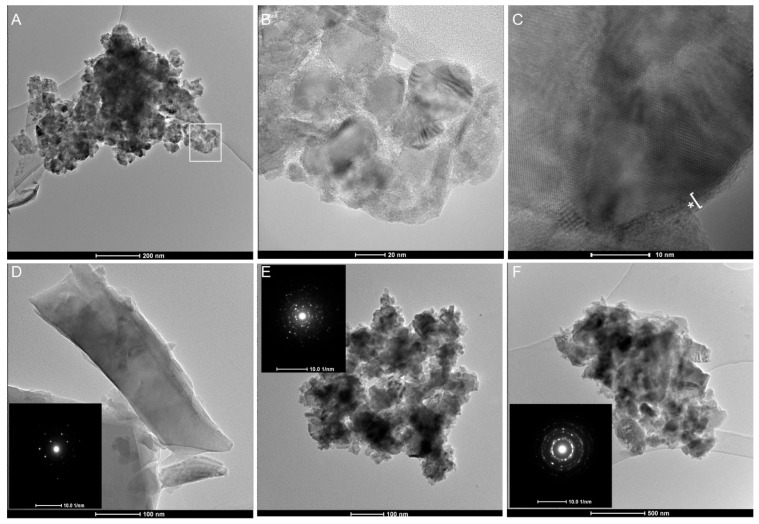
Transmission Electron Microscopy (TEM) of gQ-n3 after dispersion in ultrapure water and probe sonication. Large agglomerates of 20–30 nm quartz nanoparticles are evidenced at low magnification (**A**). The particles in the highlighted white square are imaged at higher magnification (**B**). High-resolution image of a portion of the agglomerated quartz (**C**) highlights crystalline core, with several diffraction planes visible, and the amorphous external layer (indicated by the asterisk) formed during high-energy milling. A larger submicrometric highly crystalline quartz particle ((**D**), SAED in the inset). Nanometric agglomerates of milled quartz at low magnification and their corresponding large-field SAED evidenced multiple reflections and rings that indicate a nanometric size of the primary particles ((**E**,**F**), SAED in the inset).

A further investigation on the relationship between the crystal domains and amorphization was carried out with HR-TEM (Figure 6). TEM analysis of the finest milled sample (gQ-n3) showed the presence of agglomerated nanoparticles (Figure 6A), composed of 20–30 nm primary crystalline domains (Figure 6B), which are surrounded by a ca. 5 nm amorphous layer (Figure 6C, asterisk). Surface amorphization is less evident on larger submicrometric particles, in which crystal lattice appears to be more preserved (Figure 6D). Several interference fringes, due to the overlap of differently oriented particles and relative lattice domains, were observed (Figure 6C). In particular, the 100, 011, and 101 reticular planes were individuated with a negligible dimensional shift between the experimental value and the reported literature value (Figure S3). Bimodal crystal domains resolved by Rietveld analysis were confirmed by the single area electron diffraction (SAED) images obtained from submicrometric particles (Figure 6D, inset). Moreover, in these cases, the diffraction pattern obtained indicated the occurrence of single crystals of alpha-quartz. When dense agglomerates of nanometric crystallites/particles were subjected to electron diffraction (Figure 6E,F), multiple reflections and rings confirmed the nanometric size of the gQ-n3 crystallites, as was evidenced by SEM and Rietveld analysis. This crystallinity analysis confirmed the mechanism of amorphization of the quartz particles already hypothesized in the literature and confirmed that the quantity of amorphous and the distribution of the crystalline domains are strongly linked to the comminution parameters. Our data suggest that the nanometric silica particles that occur in industrial samples may partially preserve a crystalline core.

### 2.3. Nanoquartz Particles Form Clusters in Biological Media

**Figure 7:**
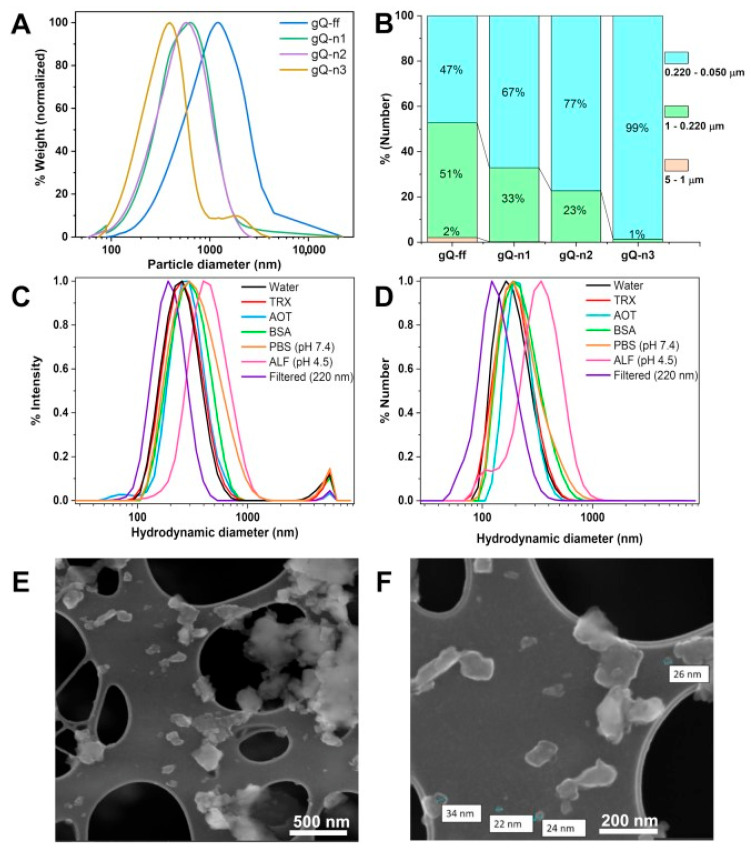
Size distribution of the fine-fractured quartz (gQ-ff) and nanoquartz samples (gQ-n1, gQ-n2, and gQ-n3) that were dispersed in water and probe-sonicated. Size distribution of the four samples analyzed by DCS, expressed as relative wt.% (**A**). Variation of the particle population in the micrometric, submicrometric, and nanometric domains, reported as relative number of particles detected by DCS (**B**). Hydrodynamic size expressed as relative intensity distribution (**C**) and relative number distribution of particles (**D**) of gQ-n3 dispersed in water (with or without post dispersion filtering through a 220 nm porous membrane), and in different biologically relevant media, i.e., Triton X (TRX), dioctyl sulfosuccinate sodium salt (AOT), bovine serum albumin (BSA), phosphate buffered saline (PBS, 10 mM, pH 7.4), artificial phagolysosome fluid (ALF, pH 4.5). Analysis was performed by DLS. FE-SEM micrographs of gQ-n3 after filtration through 220 nm pore size filter (**E**,**F**).

The particle size distribution of the nanoquartz samples dispersed in water was assessed by Differential Centrifugal Sedimentation (DCS) after probe sonication and reported as normalized weight % (Figure 7A and Table 2). For all samples, nanoparticles resulted agglomerated or aggregated in larger clusters, which could not be efficiently separated by probe sonication in water. However, a marked reduction in the average particle sizes paralleled the milling energies in the following order: gQ-ff > gQ-n1 ≈ gQ-n2 > gQ-n3, with the modal hydrodynamic diameters at 1500, 600, and 400 nm, respectively (Figure 7A).

When the DCS data were analyzed on a per number basis (Figure 7B), the dramatic size reduction from gQ-ff to gQ-n3 was further highlighted, and the clear nanometric character of the gQ-n samples was evidenced by the overwhelming number of particles in the nanodomain. Consistent with the electron microscopy observations and specific surface area measurements, the large tendency to aggregate/agglomerate prompted the primary quartz nanometric particles to form larger clusters in the aqueous medium. To test the strength of the interparticle forces that induce the clustering of the nanoquartz, the particles were dispersed in the presence of several surfactants, namely Triton X (TRX), dioctyl sulfosuccinate sodium salt (AOT), and bovine serum albumin (BSA), and simulated biological fluids (SBF), namely PBS pH 7.4 and ALF pH 4.5. The hydrodynamic size was assessed by DLS. The data obtained for gQ-n3 are reported in Figure 7C,D and Table 3, while the results for gQ-n1 and gQ-n2 are reported in Supplementary Figure S2.

Neither the surfactants nor SBF promoted the disaggregation of the three gQ-ns. Indeed, the nanoquartz particle size distribution (PSD) in TRX, AOT, and BSA overlapped that recorded in water, and a slight increase in agglomeration was even observed in the SBF. In particular, ALF induced the formation of the largest agglomerate, possibly due to the more acidic pH (pH 4.5), which decreases the quartz’s negative surface charge and the degree of electrostatic repulsion among the particles [45]. Only by applying a mechanical cutoff filter with pores of 220 nm did the size distribution of gQ-n3 shift toward lower hydrodynamic diameters. A smaller PSD was accompanied by a significant weight loss for the sample. gQ-n1 and gQ-n2 followed similar trends, as reported in Supplementary Figure S2.

FE-SEM analysis on filtered gQ-n3 (Figure 7E,F) clearly evidenced the presence of primary silica nanoparticles particles (diameter ca. 20–40 nm) and small clusters in the 100–200 nm range. This finding confirms the occurrence of the nanometric particles that were described by XRPD modeling, which could not be statistically measured by DCS and DLS analyses due to the strong aggregation of the nanoquartz in aqueous media. Even though the presence of primary isolated particles confirms the small size of these finely fractured samples, as was anticipated by the high SSA, it appears clear that only a very small part of the gQ-n samples can be disaggregated, suggesting that electrostatic interactions, even in physiological media, are strong enough to keep the particles together. This suggests that the nanometric particles that adhere to the larger particles in industrial quartz dusts are not going to be easily dispersed in physiological media, discouraging the idea that the nanometric fraction can be significantly disaggregated following dust inhalation.

### 2.4. Nanoquartz Shows NFS and Membranolytic Activity

**Figure 8:**
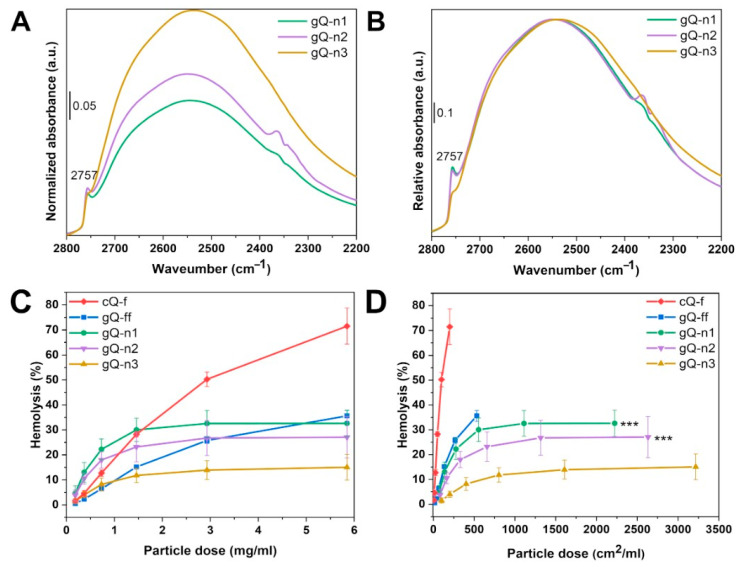
Surface silanol distribution of nanoquartz samples (gQ-n1, gQ-n2, and gQ-n3). Transmittance FTIR spectra in the 2200–2800 cm^−1^ range (νSi-OD region) were collected at b.t. after H/D isotopic exchange and subsequent outgassing for 120 min. Spectra were normalized by the bulk mode and the SSA of the particles (**A**) or by the number of interacting silanols (**B**). Hemolytic activity of fine-fractured (gQ-ff) and nanoquartz samples (gQ-n1, gQ-n2, and gQ-n3), reported as function of the particle mass (**C**) or particle exposed surface area (**D**). A mined fractured quartz (cQ-f) was used as positive reference particle for the test. Data are mean ± s.d. of three independent experiments; *p* values of gQ-n1 and gQ-n2 compared to gQ-n3 determined by two-way ANOVA followed by Tukey’s post hoc test (mean effect): *** *p* < 0.001.

To identify the surface silanol species of the nanoquartz samples (gQ-n1, gQ-n2, and gQ-n3), IR spectroscopy was carried out. Quartz particles pressed into self-supported pellets were submitted to an H/D isotopic exchange that allows for the analysis of the surface species without interference from the bulk species. The recorded spectra in the ν(Si-OD) region (2800–2200 cm^−1^) are reported in Figure 8A,B. In Figure 8A, the spectra were normalized by the bulk modes and the SSA of the particles. All three nanoquartz samples exhibited a broad band centered at ca. 2550 cm^−1^, which evidenced the presence of different strongly interacting silanol families [9,46]. The differences in the intensity of this band correlated with the differences in the SSA of the samples. Moreover, a narrow band at 2757 cm^−1^ was detected, which has been assigned to the presence of the NFS family [11]. To highlight the differences in the NFS populations of the three nanoquartz samples, the spectra were normalized by the maximum intensity of the interacting silanol band (Figure 8B). The intensities of the NFS band for gQ-n1 and gQ-n2 were almost superimposable. Conversely, the NFS band for gQ-n3 exhibited a relative lower intensity than the other two samples, with respect to the interacting silanols families. In the three samples, the NFS band at ca. 2758 cm^−1^ represented about 1.6% of the total silanols for gQ-n1, 1.5% for gQ-n2, and 0.6% for gQ-n3. In general, the silanol profiles observed for the gQ-n samples were qualitatively similar to the profiles observed for the commercial quartz flour (cQ-f) and for the pyrogenic amorphous silica particles of a similar size [11,12].

As membranolysis is strongly held to represent the molecular initiating event (MIE) in quartz inflammation [8], we assessed the capacity of the nanoquartz particles to cause RBC membrane damage using the hemolysis test. This test probes the interaction between the silica surface and cell membrane, and the RBC lysis correlated well with the silica inflammatory activity [47]. The hemolytic activity of the nanoquartz was reported as a function of both the particle mass (Figure 8C) and surface area (Figure 8D) and was compared with the activity of the nanoquartz precursor (gQ-ff) and the commercial quartz flour (cQ-f). When compared on a per mass basis (Figure 8C), gQ-ff and the nanoquartz samples were less hemolytic than cQ-f at the highest doses, but gQ-n1 and gQ-n2 were more hemolytic than all the other samples tested, at lower doses. As cQ-f has a much lower SSA than the gQ-ff and gQ-n samples, the hemolytic activity of this set of samples is possibly related to the physicochemical properties of quartz that are not directly related with the surface area exposed by the particles. In fact, when the hemolytic activity is expressed as a function of the exposed surface area (Figure 8D), the same trend was observed both at high and low doses, suggesting an inverse relationship between the surface area (i.e., the decrease in particle size) and the hemolytic effect (cQ-f >> gQ-ff > gQ-n1 ≈ gQ-n2 > gQ-n3). For the nanoquartz, this trend correlated well with the relative number of NFS (Figure 8B), suggesting that NFS are directly involved in the interaction with cell membranes, as has been already demonstrated for micrometric quartz [11]. The relative number of NFS is not, however, able to fully explain the inverse trend to SSA, as described in Figure 8C. In fact, the three nanoquartz samples exhibit a similar absolute number of NFS (Figure 8A), but the NFS density (number of NFS per nm^2^) is defined by the surface area and is reported to vary in the following order: gQ-n1 ≈ gQ-n2 > gQ-n3. This semi-quantitative observation might explain why the nanoquartz hemolytic effect shows such a prominent plateau at a relatively low concentration. We can speculate the each nanoquartz crystal has a finite binding capacity toward the RBC membrane phospholipids, which depends on the density of NFS and the contact area between the quartz and RBC. In fact, assuming the nanoquartz–RBC interaction can be reduced to a classical “contact of a sphere with a diameter D and a flat plate” problem, we can speculate that the diameter of the contact area (d_A_) is directly proportional to the diameter of the sphere (d_S_). Hence, the lower the quartz particle diameter (d_S_) is, the lower the contact area (d_A_), which is available to establish the molecular interaction between NFS and phospholipids, is.

Furthermore, the lower the nanoquartz diameter is, the lower the particle mass will be, if we consider a constant density. If we imagine that the RBC membrane is disrupted if a sufficient force is applied, we can speculate about an interaction model that takes into consideration that, given an equal number of molecular interactions established between the membrane and the quartz, the higher the mass of the particle is, the higher deformation force applied on the membrane will be. This simplified yet practical model also holds for highly hemolytic silica nanoparticles [41], in which the low contact area is likely compensated for by the high density of the NFS sites (>6% of the total silanols) [11].

## 3. Materials and Methods

### 3.1. Quartz Particles

All the reagents were purchased from Merck, in an analytical grade. Sodium metasilicate pentahydrate was purchased with a purity ≥95%, and CO_2_ gas was purchased by Sapio, Monza, Italy. As-grown quartz crystals (gQ) were obtained by hydrothermal synthesis following a procedure previously described [42], with minor modifications. A 25% (*w*/*w*) sodium metasilicate pentahydrate aqueous solution was polymerized using gaseous CO_2_ until gel formation (pH~11). Growth runs were performed in polytetrafluoroethylene liner sealed into steel autoclaves at 210 °C and under autogenic pressure for 72 h. The crystallinity of each synthetic lot was verified by X-ray powder diffraction (XRPD) analysis, and the morphology was verified by Field Emission Scanning Electron Microscopy (FESEM) analysis.

To prepare nanoquartz, a finely fractured quartz precursor (gQ-ff) was prepared from gQ (1.5 g) with a planetary ball mill (Pulverisette 6; Fritsch, Idar-Oberstein, Germany) in a ZrO_2_ jar (25 mL) with 41 g of ZrO_2_ balls (with a diameter of 5 mm), at 450 rpm for 1 h and with a pause of 1 min every 20 min. gQ-ff was then used for the preparation of the nanoquartz. Nanoquartz samples (gQ-n1, gQ-n2, and gQ-n3) were obtained by milling gQ-ff (1.5 g) in the same mill and jar described above. Smaller ZrO_2_ balls (diameter = 2 mm and total mass = 41 g), and 12 mL of ultrapure water, filtered through a 0.22 μm filter, were used to obtain the nanoquartz. The milling parameters of the different procedures used to prepare nanoquartz are reported in Table 1. After milling, the quartz suspension was washed with water and dried overnight in an oven at 70 °C. After milling, gQ-n3 was sedimented for 24 h. The supernatant containing the suspended particles was recovered and dried in the oven.

The commercial mineral quartz Min-U-Sil 5 (U.S. Silica, Berkeley Springs, WV, USA) was used as a positive reference particle (cQ-f) because of its well-documented membranolytic and toxic effects [48]. 

### 3.2. Morphology

Micrographs were acquired with a FE-SEM TESCAN S9000G equipped with a Schottky FEG source at various magnifications and accelerating voltages, commonly between 5 and 15 kV and 100 pA. Dry silica particles were deposited on conductive stubs and coated with gold to prevent the electron beam from charging the sample, when required. For measuring the primary particle size, FE-SEM was performed on a Cu TEM grid mounted on a conductive stub. Before the analysis, a few drops of the aqueous dispersion of the particles were deposited on a TEM grid and dried at room temperature. 

### 3.3. Specific Surface Area (SSA)

The SSA of the particles was evaluated by measuring Kr physisorption at −196 °C and applying the Brunauer, Emmett, and Teller (BET) method. The ASAP 2020 apparatus (Micromeritics, Norcross, GA, USA) was used. Before analysis, samples were outgassed at 150 °C for 2 h. 

### 3.4. Crystallinity

XRPD investigation was carried out on the dry samples, using a PW3050/60 X’Pert Powder X-Ray Diffractometer (Malvern Panalytical, Malvern, UK) in a spinner configuration to eliminate the preferential orientation of the powder (1 rotation/s). Diffractograms were collected between 5–120° (2θ), using Cu-Kα radiation at 45 kV and 40 mA, a step size of 0.015°, a time per step of 200 s, and a scan speed of 0.0106°/s. The Rietveld refinement [49] of the measured diffraction patterns was performed with Materials Analysis Using Diffraction (MAUD) software [50]. The instrumental function was determined using the LaB_6_ NIST standard (660b). 

The crystallinity was also checked by High Resolution Transmission Electron Microscopy (HR-TEM) analysis of the particles that were dispersed and deposited on a Cu grid, as described above. The instrument used was a Philips Tecnai G2 20 microscope, with a LaB_6_ source, operating at 200 keV. TEM micrographs were analyzed using the software ImageJ to individuate the d-spacing of the crystal lattice.

### 3.5. Particle Dispersion and Size Analysis

Analysis of the hydrodynamic diameter of the particles was performed by Differential Centrifugal Sedimentation (DCS, DC24000, CPS Instruments, Inc., Oosterhout, NL, USA) to reveal the differently sized subpopulation of particles in each sample. gQ-ff and gQ-n suspensions (1 mg/mL) were bath-sonicated for 15 min (35 kHz, ELMASONIC S10H, Augsburg, Germany) and probe-sonicated for 3 min (35% amplitude, pulse 0.5 s, UP200S, Hielscher, Berlin, Germany) right before the injection. For the DCS analysis, a gradient of sucrose between 2% and 8% was used, adding 0.1 mL of dodecane as a stabilizer. The instrument was calibrated with a polyvinyl chloride latex standard with a diameter of 0.263 µm before the sample injection. The disc speed used during the analysis was 8000 rpm for the gQ-ff samples and 11,000 rpm for the gQ-n samples.

Analysis of the disaggregation behavior of the gQ-n samples was assessed by Dynamic Light Scattering (DLS) using a Zetasizer Nano ZS (Malvern Instruments, Malvern, UK) instrument. To evaluate the possible disaggregation of the nanoquartz particles in aqueous biological-relevant media, particles were dispersed in phosphate-buffered saline (PBS, 10 mM, pH 7.4), artificial phagolysosome fluid (ALF, pH 4.5) [51], and bovine serum albumin (BSA, 2.5 mg/mL) and with surfactants. Triton X (TRX) and dioctyl sulfosuccinate sodium salt (AOT) were used at concentrations under their critical micellar concentration (CMC), 0.16 mM and 1 mM, respectively. The gQ-n samples were suspended in the dispersing agent (1 mg/mL) and bath-sonicated (FALC instruments, Treviglio, Italy) for 15 min. The suspension was diluted 1:10 with the corresponding dispersing agent and probe-sonicated (amplitude 30%, 40 W, Sonoplus HD 3100, Bandelin, Berlin, Germany) on ice for 5 min before the analysis. Moreover, an aliquot of gQ-n3 was filtered with a cellulose acetate 220 nm pore filter in ultrapure water, and its size was analyzed after sonication.

### 3.6. IR Spectroscopy

For the preparation of the self-supporting pellets, 12–15 mg of the sample were homogeneously ground, placed on a pellet die set (32 mm), and subsequently pressed at 0.8–1 tons in a manual hydraulic press (IR-Presse-25T, Maassen GmbH, Reutlingen, Germany) under atmospheric conditions. The FTIR spectra of the different quartz samples were recorded in transmission mode at beam temperature (b.t.; ca. 50 °C), using a Bruker INVENIO R spectrometer equipped with a DTGS detector at a resolution of 4 cm^−1^. The number of scans was adjusted to 64 to obtain a good signal-to-noise ratio. The powders of the silica samples were pressed into self-supporting pellets and placed in an IR quartz cell equipped with CaF_2_ windows. The cell was attached to a conventional vacuum line (residual pressure, ≤1 × 10^−3^ mbar) to perform the adsorption–desorption experiments in situ. To analyze only the surface silanols without any interference by the bulk species, the samples were submitted to an H/D isotopic exchange to convert the pristine SiOH into SiOD species via contact with heavy water vapors (D_2_O, Sigma-Aldrich; 99.90% D). The detailed H/D isotopic exchange protocol has been previously described [11]. Briefly, the samples were outgassed at b.t. for 120 min. Then, the samples were put in contact with D_2_O vapors at b.t. (ca. 20 mbar) for 15 s and subsequently outgassed for 1 min. This step was repeated until spectral invariance. Finally, the samples were outgassed at b.t. for 120 min. The collected spectra of the different samples were normalized to the intensity of the pattern in the 1720–2100 cm^−1^ region due to both the bulk modes of silica and the SSA. This allowed, on one hand, to render the differences in intensity as independent from the differences in the thickness of the pellets and, on the other hand, to perform a comparative analysis of the intensity of the surface species.

### 3.7. Membranolytic Activity

To assess the membranolytic activity of the particles, red blood cells (RBCs) were used as a simple model for the non-phagocytic cells, using a protocol previously described [12,47]. Samples were dispersed at the initial concentration of 6 mg/mL in 10 mM PBS and bath-sonicated for 15 min (FALC instruments), just before testing. Serial dilutions of the starting dispersion were performed according to the final doses used for the experiments.

### 3.8. Statistical Analysis

Statistical parameters, including the number of independent experiments and statistical significance, are reported in the figures and figure legends. Unless otherwise stated, data are mean ± s.d. (standard deviation) of three independent experiments. Normally distributed data were analyzed by two-way ANOVA followed by a Tukey’s post hoc test. A 95% confidence interval was used. Differences with *p* < 0.05 were considered statistically significant. Statistical analysis was performed with the GraphPad Prism 9 software.

## 4. Conclusions

In this study, a nanometric quartz was prepared from a pure synthetic α-quartz by high-energy ball milling. Its toxicity-relevant features and its membranolytic activity were investigated, and the nanoquartz was proven to share key surface properties with toxic quartz crystals of a larger size. The energy transfer to the sample during milling exceeded by far the energy of conventional industrial processing, but the top-down approach adopted allowed for obtaining quartz nanoparticles in 1.5 g batches and in a conveniently short time. The aim of this work was indeed to generate larger quantities of nanometric silica particles with a similar size range and biologically active surfaces that are detected on industrially produced quartz dusts. Our quartz nanoparticles showed dimensional and adhesion properties that can be compatible with the quartz nanoparticles generated during standard workplace operations. They exhibited very strong interparticle adhesion forces, a specific surface area greater than 50 m^2^/g, a primary particle size in the nanometric range, and a negligible number of micrometric particles. Size distribution of the crystallite domains by Rietveld modeling evidenced the occurrence of a bimodal distribution, with two populations of nanometric and submicrometric crystal domains. During the milling procedure, some nanometric crystalline particles were progressively amorphized, likely due to local thermal effects, but a relevant number of crystalline nanometric particles were preserved, as evidenced by TEM. The milled quartz particles showed a strong tendency toward clustering in biologically relevant media, and the nanoquartz could not be deagglomerated by surfactants and sonication, suggesting that strongly interacting aggregates are formed, which also persisted in biological media. However, the strong aggregation does not hamper the bioavailability of the nanoquartz aggregates, which largely remains in the respirable size range. Furthermore, the interparticle electrostatic forces that promote aggregation in our samples might well explain the difficulties in separating the nanometric fraction from the micrometric one in industrially generated quartz dusts. Nearly free silanols (NFS), the surface moiety held responsible for the membranolytic activity of silica, were observed on the nanoquartz, and their relative number correlated well with the ability of the nanoquartz to cause red blood cell membrane lysis. This study provides a new feasible route for the top-down preparation of nanometric quartz with a controlled size and toxicological properties and allows us to define, for the first time, the physico-chemical properties of a nanometric quartz particle that is compatible with the nanometric fraction of an industrial quartz dust. Further investigations will use these samples to assess the relevance of nanoquartz in the overall toxic effect of crystalline silica.

## Figures and Tables

**Table 1:** Milling parameters and specific surface area of the quartz particles.

	Quartz	Origin	Milling Speed (rpm)	Milling Time (Hours)	Ball Diameter (mm)	SSA (m^2^/g) ^b^
A	gQ	Synthesis	/	/	/	0.1
B	gQ-ff	gQ dry milling	450	1	5	10
C1	gQ-n1 ^a^	gQ-ff wet milling	450	1	2	38
C2	gQ-n2 ^a^	gQ-ff wet milling	550	1	2	45
C3	gQ-n3 ^a^	gQ-ff wet milling	550	2	2	55

^a^ Wet milled, with 12 mL of water. ^b^ Measured by the BET method, using Kr as adsorbing gas.

**Table 2:** Particle size distribution (PSD) of the milled quartz samples that were dispersed in water, probe-sonicated, and measured by DCS.

Quartz	Peak (µm)	Range % (22–5.0 µm)	Range % (5.0–1.0 µm)	Range % (1.0–0.22 µm)	Range % (0.22–0.050 µm)
gQ-ff	1.45	23.1	46.37	24.75	1.17
gQ-n1	0.635	0.0	13.42	73.06	6.97
gQ-n2	0.574	0.0	12.27	76.44	10.49
gQ-n3	0.392	0.0	7.42	72.43	19.93

**Table 3:** Particle size distribution of nanoquartz (gQ-n3) dispersed in biologically relevant media measured by DLS.

Medium	Z-Average (nm) ± s.d. ^a^	Peak (nm) ± s.d. ^a^ Intensity	Peak (nm) ± s.d. ^a^ Number
H_2_O	276.2 ± 16.1	264.8 ± 14.0	198.3 ± 6.6
H_2_O + TRX	280.4 ± 13.9	274.8 ± 20.7	199.0 ± 14.8
H_2_O + AOT	298 ± 4.5	274.8 ± 52.9	175.9 ± 81.3
H_2_O + BSA	321.6 ± 6.7	315.7 ± 16.4	238.0 ± 7.0
PBS	330.9 ± 3.2	366.9 ± 33.4	243.0 ± 2.0
ALF	435.0 ± 26.9	470.0 ± 48.8	345.2 ± 62.9
Filtered (220 nm-pores)	246.8 ± 20.7	203.0 ± 10.7	144.2 ± 19.5

^a^ Standard deviation (s.d.) calculated on two independent experiments.

## Data Availability

The raw data were generated at the University of Turin, Italy, and the University of Namur, Belgium. The derived data supporting the findings of this study are available from F.T. upon reasonable request.

## Supplementary Materials

The following are available online at https://www.mdpi.com/article/10.3390/ijms232315425/s1.

## Abbreviations

ALF	artificial phagolysosome fluid
AOT	dioctyl sulfosuccinate sodium salt
BSA	bovine serum albumin
b.t.	beam temperature
DCS	differential centrifugal sedimentation
NFS	nearly free silanols
RCS	respirable crystalline silica
SAED	single area electron diffraction
TRX	Triton X
