# Supplementary material for: Top-Down Preparation of Nanoquartz for Toxicological Investigations
Source: Int J Mol Sci. 2022 Dec 6;23(23):15425. doi: 10.3390/ijms232315425 (PMC9738116; doi:10.3390/ijms232315425)
Supplement: Supplementary file 1 [file ijms-23-15425-s001.zip › ijms-2052368-supplementary.pdf]

Supplementary Material

Chiara Bellomo [1,2], Cristina Pavan [1,2,3], Gianluca Fiore [1,4], Guillermo Escolano-Casado [1,4], Lorenzo Mino [1,4] and Francesco Turci [1,2,4,*]

[1] Department of Chemistry, University of Turin, 10125 Turin, Italy
[2] "G. Scansetti" Interdepartmental Centre for Studies on Asbestos and Other Toxic Particulates, University of Turin, 10125 Turin, Italy
[3] Louvain Centre for Toxicology and Applied Pharmacology, Université catholique de Louvain, 1200 Brussels, Belgium
[4] Nanostructured Interfaces and Surfaces Interdepartmental Centre, University of Turin, 10125 Turin, Italy
* Correspondence: francesco.turci@unito.it; Tel.: +39-011-6707566

This document includes:

1. FE-SEM micrographs of gQ-n1, gQ-n2 and gQ-n3;
2. DLS analysis of gQ-n1 and gQ-n2;
3. TEM micrographs of gQ-n3;
4. SEM-EDS analysis of gQ before milling;
5. Full range IR spectra (1300 cm^{-1} – 4000 cm^{-1}) of gQ-n1, n2, and n3 before and after D_2O exchange.

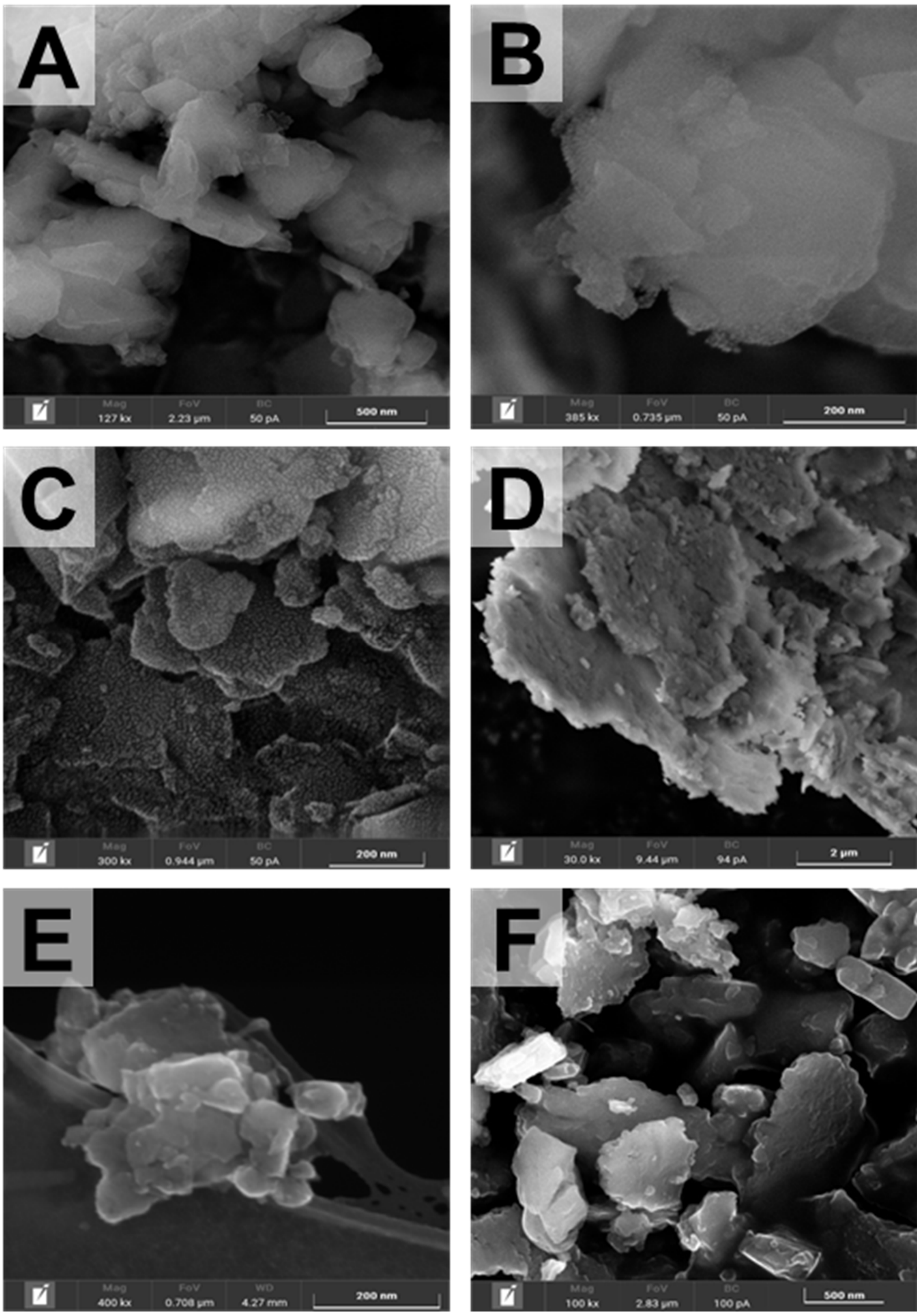

Figure S1. SEM micrographs of **(A-B)** gQ-n1, **(C-D)** gQ-n2, **(E-F)** gQ-n3 at various magnification (reported on the micrographs).

gQ-n1

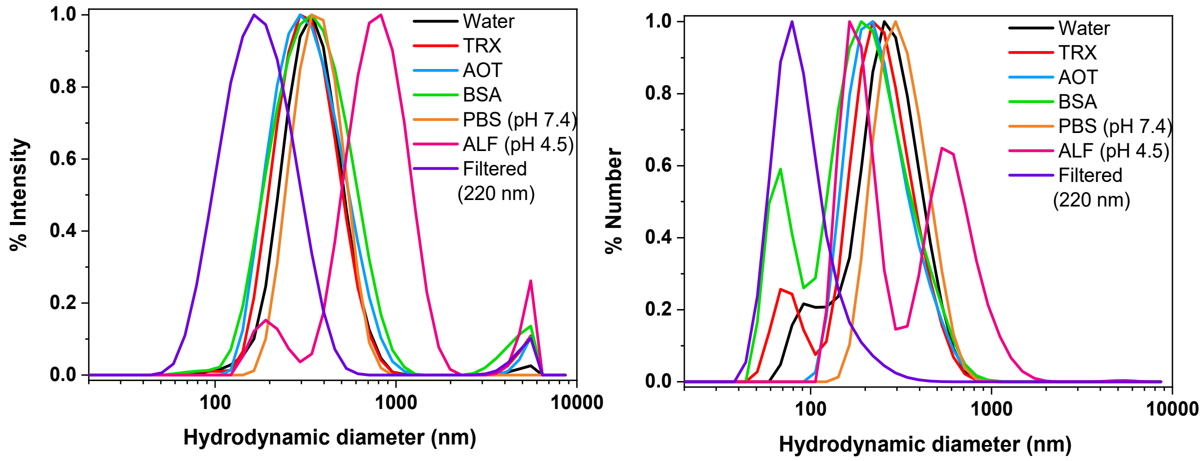

gQ-n2

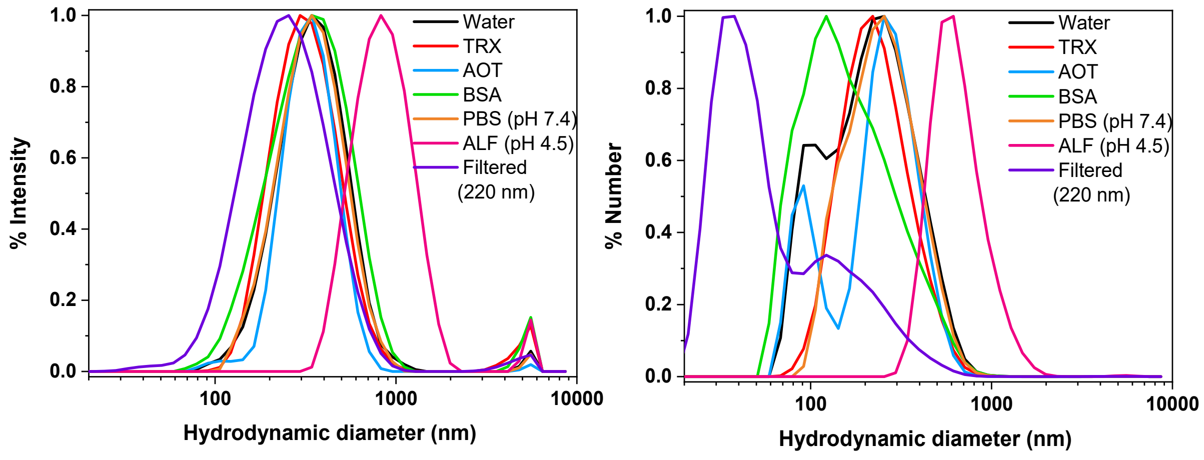

Figure S2. Hydrodynamic size reported in intensity-distribution and number-distribution of gQ-n1 and gQ-n2 measured by DLS analysis. Particles were dispersed in water and in different biologically relevant media, i.e., Triton X (TRX), dioctyl sulfosuccinate sodium salt (AOT), bovine serum albumin (BSA), phosphate buffered saline (PBS, 10 mM, pH 7.4), artificial phagolysosome fluid (ALF, pH 4.5), and filtered through 200-nm pore size filter.

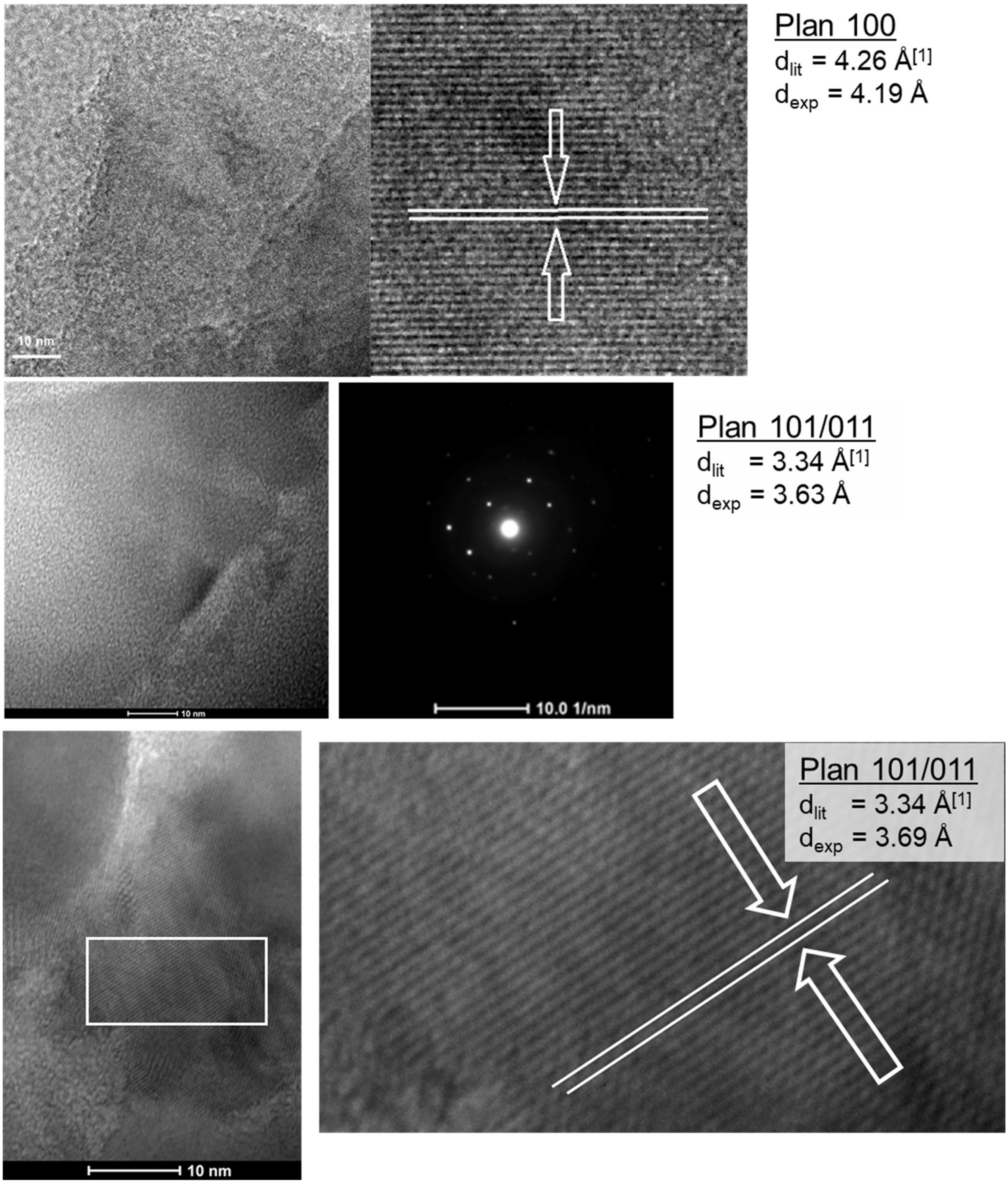

Figure S3: Elaboration of TEM data and individuation of the crystal planes of gQ-n3.

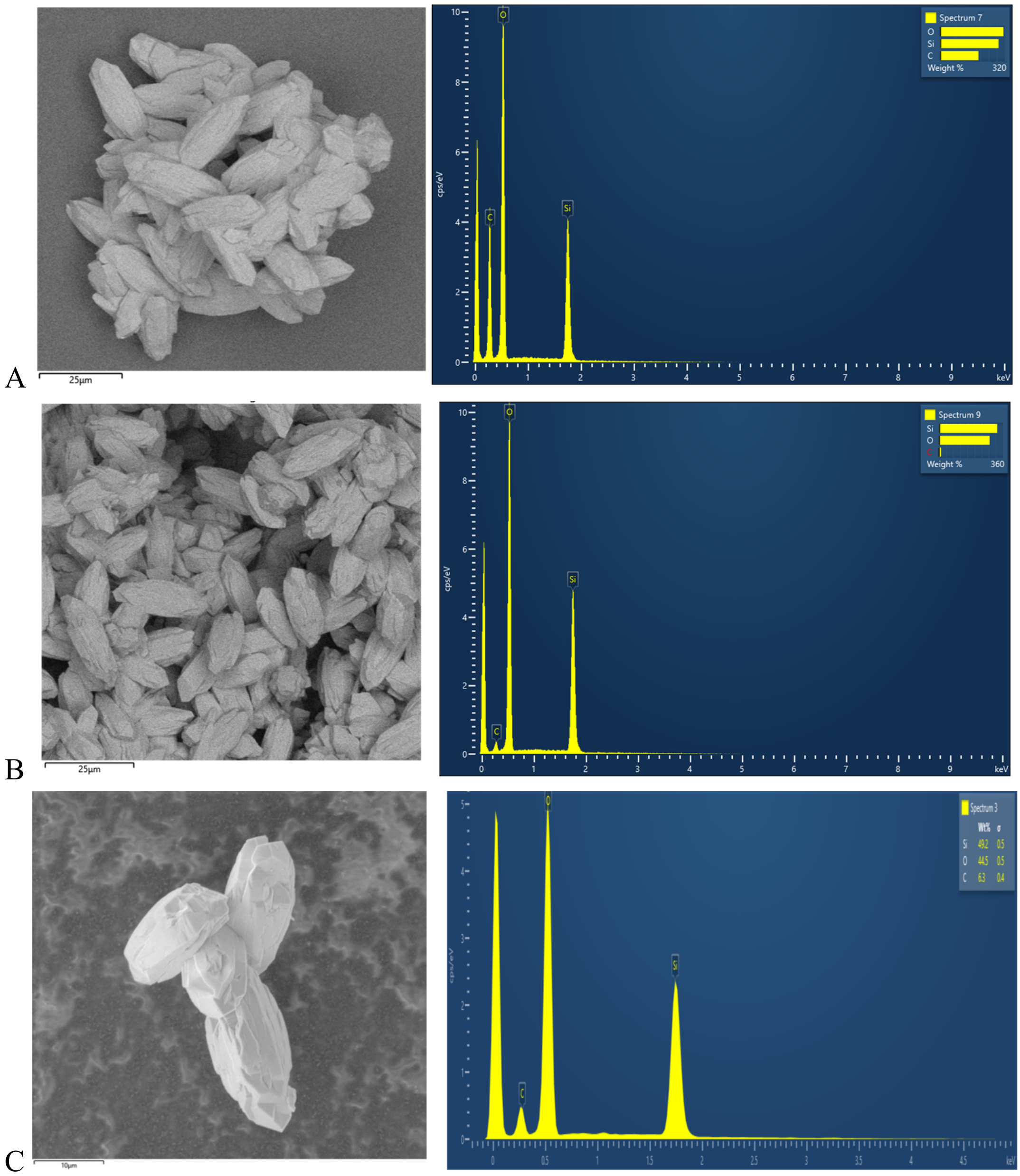

Figure S4: SEM-EDS images (BSE electrons) analysis of some batches of the synthetized gQ samples. In all cases, only Si and O were detected. The presence of C is due to the SEM conductive tape.

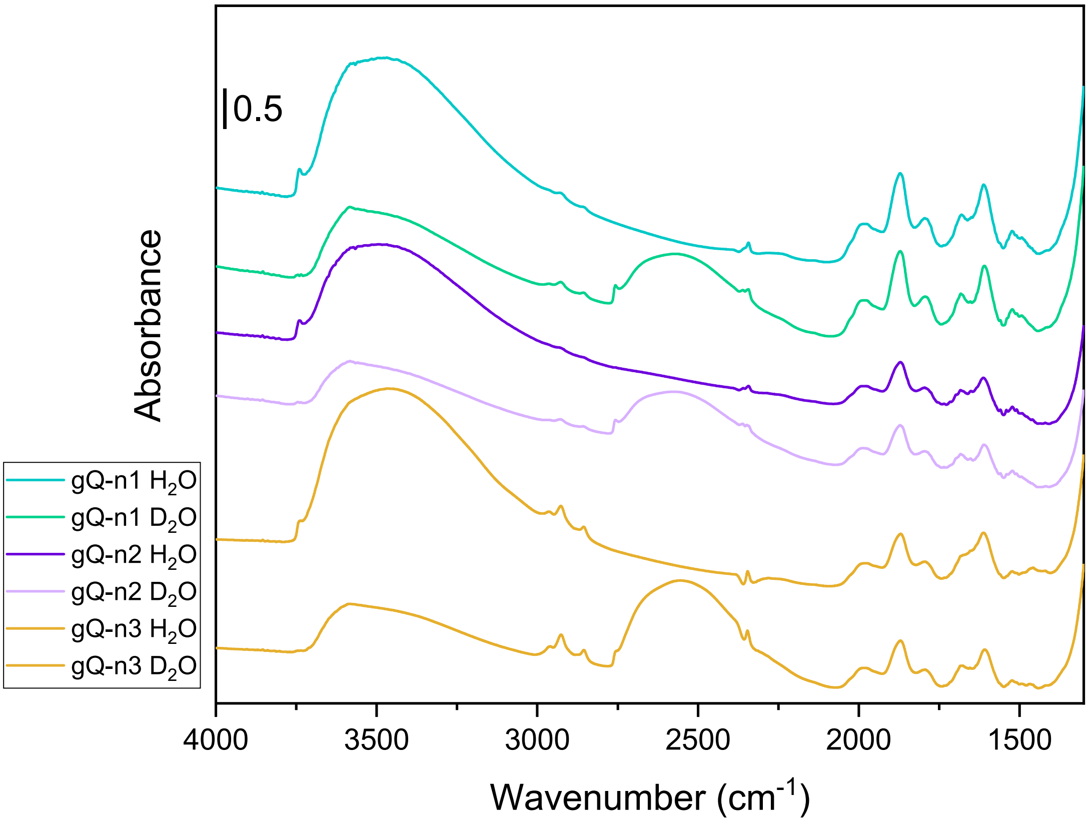

Figure S5: Full range IR spectra of gQ-n1, n2, and n3 before and after D_2O exchange.
